# Concordance of human equilibrative nucleoside transporter‐1 expressions between murine (10D7G2) and rabbit (SP120) antibodies and association with clinical outcomes of adjuvant chemotherapy for pancreatic cancer: A collaborative study from the JASPAC 01 trial

**DOI:** 10.1002/cnr2.1507

**Published:** 2021-07-29

**Authors:** Yukiyasu Okamura, Narikazu Boku, Paula Ghaneh, William Greenhalf, Satoru Yasukawa, Hiroto Narimatsu, Akira Fukutomi, Masaru Konishi, Soichiro Morinaga, Hirochika Toyama, Atsuyuki Maeda, Yasuhiro Shimizu, Shoji Nakamori, Naohiro Sata, Keisuke Yamakita, Amane Takahashi, Wataru Takayama, Ryuzo Yamaguchi, Moriaki Tomikawa, Akio Yanagisawa, John P. Neoptolemos, Katsuhiko Uesaka

**Affiliations:** ^1^ Division of Hepato‐Biliary‐Pancreatic Surgery Shizuoka Cancer Center Hospital Nagaizumi Japan; ^2^ Division of Gastrointestinal Medical Oncology National Cancer Center Hospital Tokyo Japan; ^3^ Department of Molecular and Clinical Cancer Medicine University of Liverpool Liverpool UK; ^4^ Department of Surgical Pathology Kyoto Prefectural University of Medicine Kyoto Japan; ^5^ Department of Pathology Japanese Red Cross Kyoto Daini Hospital Kyoto Japan; ^6^ Cancer Prevention and Control Division Kanagawa Cancer Center Yokohama Japan; ^7^ Division of Gastrointestinal Oncology Shizuoka Cancer Center Shizuoka Japan; ^8^ Division of Hepato‐Biliary‐Pancreatic Surgery National Cancer Center Hospital East Kashiwa Japan; ^9^ Division of Gastrointestinal Surgery Kanagawa Cancer Center Yokohama Japan; ^10^ Division of Hepato‐Biliary‐Pancreatic Surgery Kobe University Kobe Japan; ^11^ Division of Surgery Ogaki Municipal Hospital Ogaki Japan; ^12^ Division of Gastrointestinal Surgery Aichi Cancer Center Hospital Nagoya Japan; ^13^ Division of Surgery National Hospital Organization Osaka National Hospital Osaka Japan; ^14^ Division of Gastrointestinal Surgery Jichi Medical University Shimotsuke Japan; ^15^ Division of Metabolism and Biosystemic Science, Department of Medicine Asahikawa Medical University Asahikawa Japan; ^16^ Division of Gastrointestinal Surgery Saitama Cancer Center Saitama Japan; ^17^ Division of Gastrointestinal Surgery Chiba Cancer Center Chiba Japan; ^18^ Division of Surgery Kasugai Municipal Hospital Kasugai Japan; ^19^ Division of Surgery Tochigi Cancer Center Utsunomiya Japan; ^20^ Department of Pathology Japanese Red Cross Kyoto Daiichi Hospital Kyoto Japan; ^21^ Department of General Visceral and Transplantation Surgery, University of Heidelberg Germany

**Keywords:** 10D7G2, gemcitabine, human equilibrative nucleoside transporter‐1, pancreatic cancer, SP120

## Abstract

**Background:**

Expression of human equilibrative nucleoside transporter‐1 (hENT1) is reported to predict survival of gemcitabine (GEM)‐treated patients. However, predictive values of immunohistochemical hENT1 expression may differ according to the antibodies, 10D7G2 and SP120.

**Aim:**

We aimed to investigate the concordance of immunohistochemical hENT1 expression between the two antibodies and prognosis.

**Methods:**

The subjects of this study were totally 332 whose formalin‐fixed paraffin‐embedded specimens and/or unstained sections were obtained. The individual H‐scores and four classifications according to the staining intensity were applied for the evaluation of hENT1 expression by 10D7G2 and SP120, respectively.

**Results:**

The highest concordance rate (79.8%) was obtained when the cut‐off between high and low hENT1 expression using SP120 was set between moderate and strong. There were no correlations of *hENT1* mRNA level with H‐score (p = .258). Although the *hENT1* mRNA level was significantly different among four classifications using SP120 (p = .011), there was no linear relationship among them. Multivariate analyses showed that adjuvant GEM was a significant predictor of the patients with low hENT1 expression using either 10D7G2 (Hazard ratio [HR] 2.39, p = .001) or SP120 (HR 1.84, p < .001). In contrast, agent for adjuvant chemotherapy was not significant predictor for the patients with high hENT1 expression regardless of the kind of antibody.

**Conclusion:**

The present study suggests that the two antibodies for evaluating hENT1 expression are equivalent depending on the cut‐off point and suggests that S‐1 is the first choice of adjuvant chemotherapy for pancreatic cancer with low hENT1 expression, whereas either S‐1 or GEM can be introduced for the pancreatic cancer with high hENT1 expression, no matter which antibody is used.

## 1 INTRODUCTION

Gemcitabine (GEM) is a key drug of pancreatic cancer (PC).^1^, ^2^, ^3^, ^4^ Expression of human equilibrative nucleoside transporter‐1 (hENT1) has been reported to be related with sensitivity to GEM in several cancer types including PC.^5^, ^6^, ^7^ High immunohistochemistry (IHC) expression of hENT1 in tumor tissue (hENT1^High^) is associated with better survival benefit from adjuvant GEM.^8^, ^9^, ^10^, ^11^, ^12^, ^13^, ^14^, ^15^ However, most of these results were based on the IHC using murine 10D7G2 monoclonal anti‐hENT1 antibody (Ab), which is not commercially available.^8^, ^9^, ^10^, ^11^, ^12^, ^13^, ^14^ Alternatively, the SP120 rabbit monoclonal anti‐hENT1 Ab has been developed and used to evaluate hENT1 expression, and three studies did not find consistent association between IHC expression of hENT1 using the SP120 Ab and prognosis in PC patients treated with GEM.^15^, ^16^, ^17^

Moreover, three studies comparing the 10D7G2 and SP120 Abs have been reported.^14^, ^18^, ^19^ One study found that hENT1 expression, as evaluated by IHC using tissue microarray (TMA), matched between the two Abs in two thirds of cases, and concluded that both Abs could predict prognosis in patients who received GEM as adjuvant chemotherapy.^19^ In contrast, the other study found that IHC hENT1 expression matched in half of cases, and concluded that only the 10D7G2 Ab was useful for predicting prognosis.^14^, ^18^ However, both studies used specimens collected over ≥10 years,^14^, ^18^, ^19^ and during such a long study period their outcomes have been affected by changes in treatment strategies after introduction of adjuvant chemotherapy.^20^

Previously, we reported on the association of IHC expression of hENT1 using SP120 Ab with overall survival in patients enrolled into the Japan Adjuvant Study Group of Pancreatic Cancer (JASPAC) 01 study which randomized 377 pancreatic cancer patients to receive either GEM or S‐1 after curative resection.^20^, ^21^ Interestingly, in the S‐1 arm, the median overall survival in patients with high hENT1 expression was significantly shorter than the patients with low hENT1 expression.^21^ Thus, predictive values of hENT1 IHC expression may differ according to the Abs, 10D7G2 and SP120. Furthermore, there may be some patients for whom GEM would show equivalent or better efficacy than S‐1, despite the results of the JASPAC 01 study showing significantly better prognosis of S‐1 than GEM in overall.

In the present study, we evaluated the concordance of hENT1 expression between 10D7G2 and SP120 Abs, and explored their relation to *hENT1* mRNA level and survival in subsets of patients enrolled into the JASPAC 01 study.^20^, ^21^

## 2 MATERIALS AND METHODS

### 2.1 Study population and design

This biomarker study was designed as a collaborative study of the JASPAC 01 study^21^ after the completing its final analysis. The protocol of the present study was approved by the ethics committee of the Shizuoka Cancer Center (No. 27‐22‐27‐1‐5) and the institutional review board of each participating institution. From totally 332 of all 377 patients enrolled in the JASPAC 01 study at the 24 participating institutions, we collected the unstained sections for IHC using the SP120 Ab (*n* = 326: 86.5%) and for measuring mRNA level of hENT1, and/or formalin‐fixed, paraffin‐embedded (FFPE) specimen blocks for TMA using the 10D7G2 Ab (*n* = 114: 30.2%).

**FIGURE 1:**
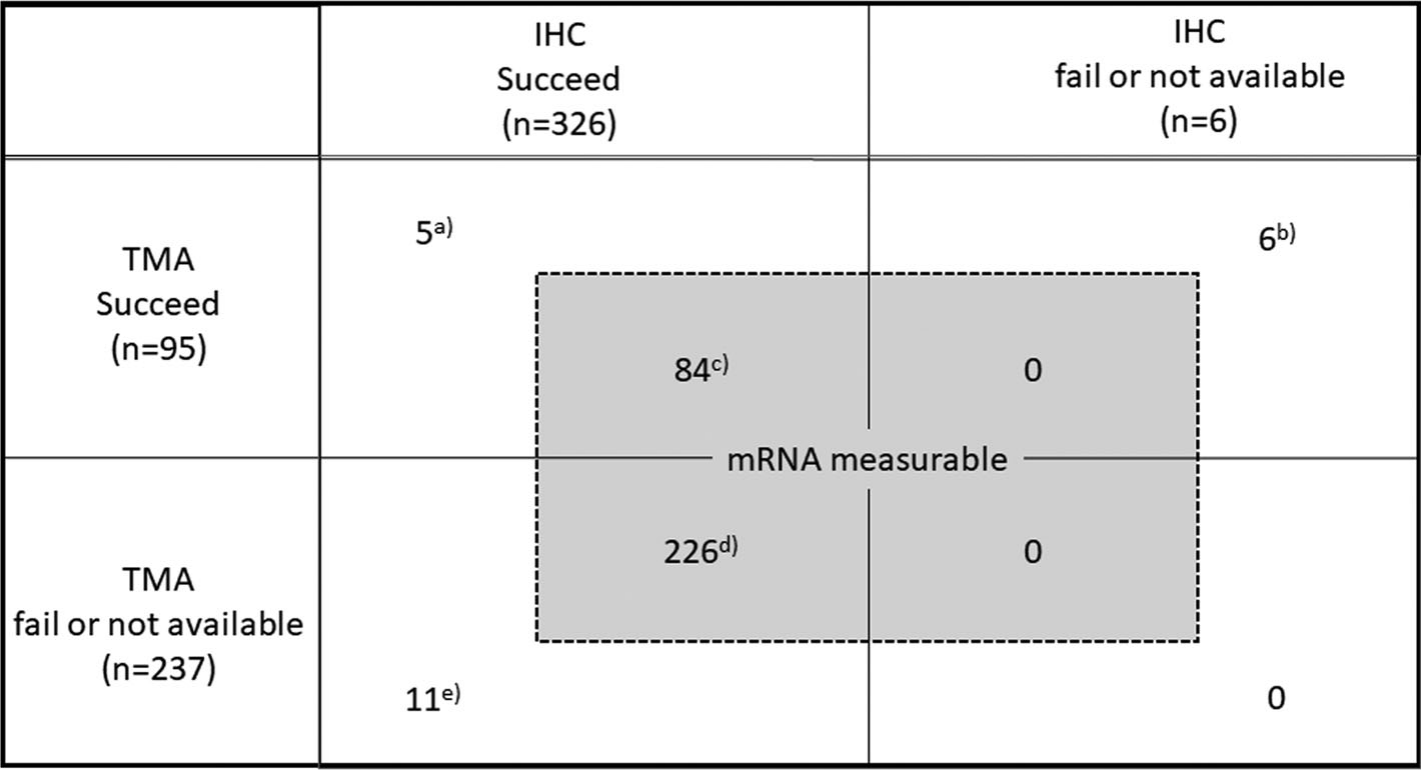
Distribution of patients for three methods assessing hENT1

Distribution of patients whose samples were evaluable for IHC, TMA, and measurable for *hENT1* mRNA level is shown in Figure 1. The concordance between the two Abs was investigated in 89 patients for whom both IHC and TMA were evaluable (Figure 1(A),(C)). The relationship of expression level of *hENT1* mRNA with IHC was investigated in 310 patients (Figure 1(C),(D)), and that with TMA was in 84 patients (Figure 1(D)).

### 2.2 TMA with the 10D7G2 Ab

The University of Liverpool UK prepared TMAs from the FFPE specimen blocks of the 114 patients and were blindly evaluated for hENT1 expression using the 10D7G2 Ab, as previously described.^11^ The cut‐off value of H‐score between high hENT1 expression (10D7G2^High^) and low hENT1 expression (10D7G2^Low^) was determined by the minimum p value approach in survival analysis.

### 2.3 IHC with the SP120 Ab

Expression of hENT1 IHC using the SP120 Ab was evaluated on unstained sections (anti‐hENT1 rabbit monoclonal Ab SP120, Roche Tissue Diagnostics Co, Ltd, Basel, Switzerland; already diluted Ab) as previously described.^21^ IHC was evaluated under light microscopy by two pathologists (SY and AY) who were blinded to all clinical information. Tumor cell immunostaining was classified into four groups according to staining intensity (Figure S1: A, absent; B, weak; C, moderate; or D, strong).

### 2.4 Reverse transcription‐polymerase chain reaction

Representative hematoxylin and eosin‐stained sections (10 μm‐thick) were reviewed by a pathologist who marked out cancer predominant areas, which were removed by macro‐dissection. RNA was extracted from the removed tumor tissue with the RNeasy FFPE Kit (Qiagen, Chatsworth, CA), and cDNA was prepared using a High Capacity cDNA Reverse Transcription Kit with RNase Inhibitor (Life Technologies, Foster City, CA) according to the manufacturer's protocol. Expression of the *hENT1* mRNA was measured using a TaqMan real‐time PCR (Life Technologies, Foster City, CA), as described previously.^22^ Expression of glyceraldehyde‐3‐phosphate dehydrogenase (*GAPDH*) mRNA was quantified in each sample and used to standardize the data. We measured the cycle threshold (*Ct*) value, which is inversely proportional to the amount of cDNA. Analysis was performed in triplicate for all samples. The expression level of each sample was shown as the value of each target divided by the *GAPDH* value.

### 2.5 Statistical analysis

Continuous variables are presented as median and range, and were compared using the Mann–Whitney *U* test or Kruskal Wallis test, as appropriate. Categorical variables were compared using the chi‐square test or Fisher's exact test, as appropriate. The cut‐off value of *hENT1* mRNA levels was explored using receiver operating characteristic curves for the overall survival and Youden's index. Overall survival rates were estimated using the Kaplan–Meier method and compared by the log‐rank test. The Cox proportional hazards model was used for univariate and multivariate analyses, and treatment arms (GEM or S‐1) and all factors found to be significant predictors of overall survival (p < .10) in univariate analysis were entered into multivariate analysis. All statistical analyses were performed using the SPSS 24.0 software package (SPSS, Inc., Chicago, IL). p < .05 (two‐tailed) was considered significant.

## 3 RESULTS

### 3.1 Patient characteristics

The clinical and pathological characteristics of the 332 patients (Figure 1(A)–(F)) in the present study was shown in Table 1. The median overall survival time after randomization was 2.16 years with GEM and 3.76 years with S‐1 (Figure S2(A)). The hazard ratio (HR) for mortality of S‐1, compared with GEM, was 0.60 (95% confidence interval [CI] 0.46–0.78, p < .001). The median relapse‐free survival time after randomization was 1.07 years with GEM and 1.88 years with S‐1 (Figure S2(B)). The HR was 0.66 (95% CI 0.51–0.85, p = .001).

**TABLE 1:** The clinical and pathological characteristics of the 332 patients according to the adjuvant chemotherapy agents in the present study

	All, *n* (%)	Gemcitabine, *n* (%)	S‐1, *n* (%)	
	332 (100)	170 (100)	162 (100)	p
Sex				.440
Male	185 (56)	91 (54)	94 (58)	
Female	147 (44)	79 (46)	68 (42)	
Age^a^	66 (34–86)	66 (44–84)	66 (34–86)	.805
ECOG performance status				.561
0	222 (67)	111 (65)	111 (69)	
1	110 (33)	59 (35)	51 (31)	
Operative procedure				.053
Pancreatoduodenectomy	224 (67)	121 (71)	103 (64)	
Distal pancreatectomy	105 (32)	46 (27)	59 (36)	
Total pancreatectomy	3 (1)	3 (2)	0 (0)	
Combined portal vein resection	96 (29)	50 (29)	46 (28)	.904
Number of dissected lymph nodes^a^	25 (1–81)	26 (2–81)	24 (1–77)	.404
Residual tumor status				.751
R0	286 (86)	145 (85)	141 (87)	
R1	46 (14)	25 (15)	21 (13)	
Primary tumor status^b^				.862
T1–T2	36 (11)	19 (11)	17 (10)	
T3–T4	296 (89)	151 (89)	145 (90)	
Regional lymph node status^b^				.302
N0	116 (35)	64 (38)	52 (32)	
N1	216 (65)	106 (62)	110 (68)	
CA19‐9				.502
≤37 U/ml	263 (79)	132 (78)	131 (81)	
>37 U/ml	69 (21)	38 (22)	31 (19)	
Pathological stage^b^				.333
IA	17 (5)	8 (5)	9 (6)	
IB	8 (2)	6 (3)	2 (1)	
IIA	90 (27)	49 (29)	41 (25)	
IIB	215 (65)	107 (63)	108 (67)	
III	2 (1)	0 (0)	2 (1)	

Abbreviation: ECOG, Eastern Cooperative Oncology Group.

^a^ Median (range).

^b^ Primary tumor status, regional lymph node status, and pathological stage according to the TNM Classification of Malignant Tumours, 6th edition.

### 3.2 Concordance of hENT1 expression between TMA with 10D7G2 and IHC with SP120

hENT1 expression was evaluable both by TMA with 10D7G2 Ab and by IHC with SP120 Ab in 89 patients (Figure 1(A),(C)). Median (range) H‐score for 10D7G2‐assessed hENT1 expression was 100 (0–186), and the cut‐off value of H‐score was determined to be 135 by the minimum p value approach in survival analysis. There were 16 patients (18%) whose H‐scores were ≥135 (10D7G2^High^), and IHC expression of hENT1 using SP120 were strong in 17, moderate in 31, weak in 31, and absent in 10 patients (Table 2).

**TABLE 2:** Correlation between IHC expression of hENT1 using SP120 Ab and H‐score (high/low) by TMA using 10D7G2 Ab

		10D7G2		
		Low	High	
SP120	Absent	15	2	17 (19%)
	Weak	27	4	31 (35%)
	Moderate	25	6	31 (35%)
	Strong	6	4	10 (11%)
		73 (82%)	16 (18%)	

Abbreviations: Ab, antibody; hENT1, human equilibrative nucleoside transporter‐1; IHC, immunohistochemistry; TMA, tissue microarray.

Correlation between IHC expression of hENT1 using SP120 and H‐score (high/low) by TMA using 10D7G2 is shown in Table 2. The highest concordance between the two evaluation methods in 2 × 2 categorization was obtained when the cut‐off between high and low IHC expression using SP120 (SP120^Low^ and SP120^High^) was set between moderate and strong (concordance rate: 79.8%). When the cut‐off between SP120^Low^ and SP120^High^ was set between weak and moderate, the concordance rate was 58.4%. The cut‐off set between absent and weak showed a concordance rate as low as 32.6%.

### 3.3 Relationship between *hENT1* mRNA level and hENT1 expression with TMA by 10D7G2 (H‐score) and with IHC by SP120

Relationship between *hENT1* mRNA level and H‐score was investigated in the 84 patients (Figure 1(C)). There were no significant associations between them (Pearson's correlation coefficient: 0.125; p = .258; Figure S3(A)) and between categorical 10D7G2 hENT1 expression (10D7G2^High^ /10D7G2^Low^) and *hENT1* mRNA level (p = .350).

*hENT1* mRNA level and hENT1 expression with IHC by SP120 were evaluable in 310 patients (Figure 1(C),(D)). The median *hENT1* mRNA expression levels (range) in tissues with strong, moderate, weak and absent IHC staining were 24.3 (3.73–228.7), 14.0 (1.02–272.1), 16.2 (1.69–160.3) and 26.2 (2.20–163.6), respectively. Although the *hENT1* mRNA expression level was significantly different among patients with four SP120 IHC expressions (*n* = 310; Kruskal Wallis test, p = .011; Figure S3(B)), there was no linear relationship between them.

### 3.4 Overall survival by 10D7G2 hENT1 expression

Among the 95 patients whose H‐score by TMA with 10D7G2 Ab were evaluable (Figure 1(A)–(C)), we compared the clinical and pathological characteristics between the patients with 10D7G2^High^ and 10D7G2^Low^ (Table S1). Although not significant, the proportions with lymph node metastasis and with low CA19‐9 level were slightly higher in 10D7G2^High^ patients in both treatment groups.

**FIGURE 2:**
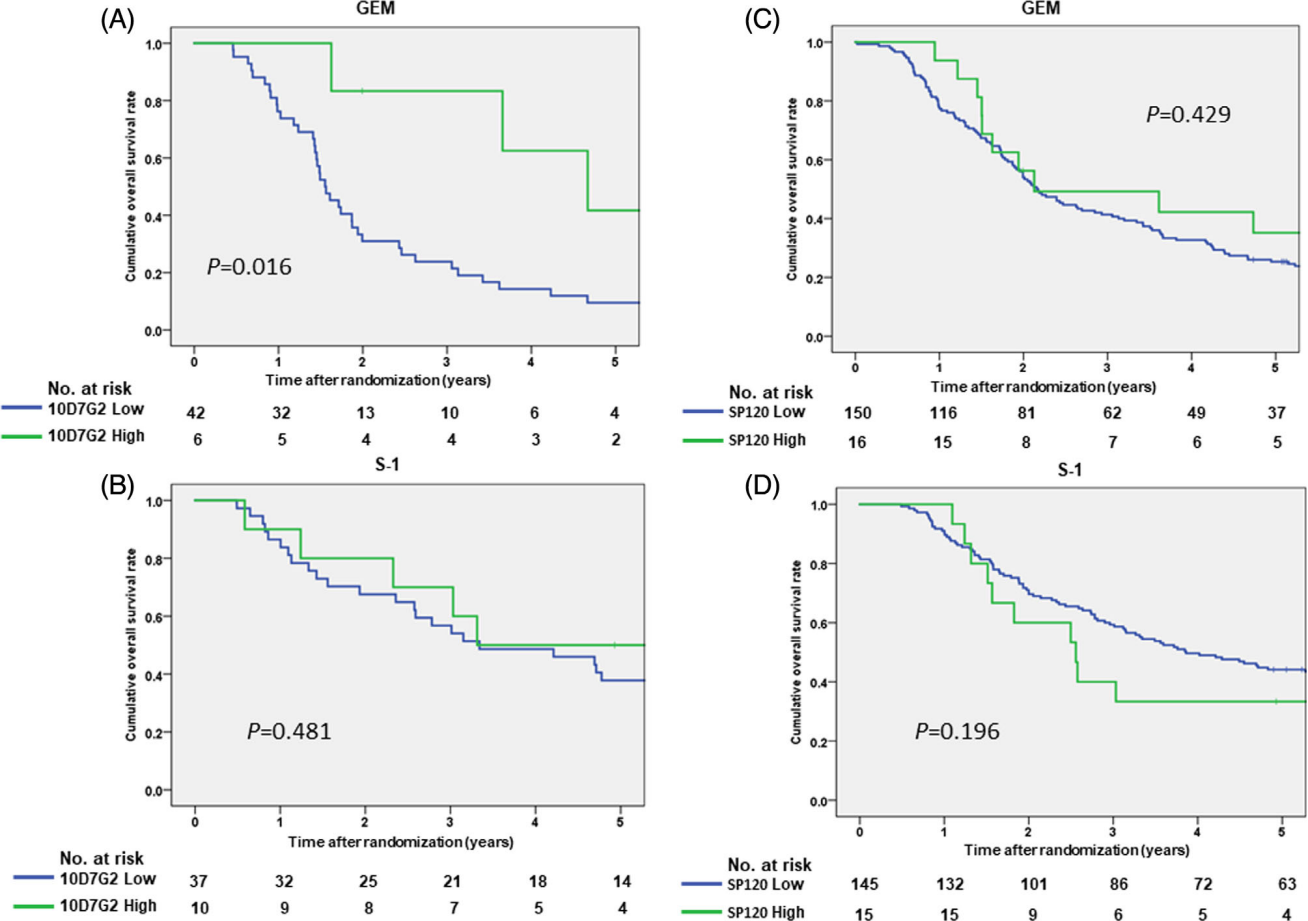
(A) Kaplan–Meier curves for overall survival in the gemcitabine arm, stratified by human equilibrative nucleoside transporter‐1 (hENT1) expression by 10D7G2. (B) Kaplan–Meier curves for overall survival in the S‐1 arm, stratified by hENT1 expression by 10D7G2. (C) Kaplan–Meier curves for overall survival in the gemcitabine arm, stratified by hENT1 expression by SP120. (D) Kaplan–Meier curves for overall survival in the S‐1 arm, stratified by hENT1 expression by SP120

Six (12.5%) of the 48 patients receiving adjuvant chemotherapy with GEM had 10D7G2^High^ expression. The GEM‐treated 10D7G2^High^ patients had significantly longer overall survival than the remaining 42 GEM‐treated 10D7G2^Low^ patients (median 4.67 versus 1.55 years, HR [10D7G2^Low^] 3.89, 95% CI 1.19–12.7, p = .016, Figure 2(A)). In contrast, among patients receiving adjuvant chemotherapy with S‐1, overall survival did not significantly differ between the 10D7G2^High^ and 10D7G2^Low^ patients (median 3.31 vs. 3.34 years, HR [10D7G2^Low^] 1.41, 95% CI 0.54–3.70, p = .481, Figure 2(B)).

Multivariate analyses showed that hENT1 expression with 10D7G2 (high vs. low) (HR 0.24, 95% CI 0.08–0.84, p = .024) and sex (male vs. female) (HR 1.90, 95% CI 1.03–3.51, p = .040) were significant predictor for the survival of the patients who received with GEM as adjuvant chemotherapy agent (Table 3). In contrast, there were no significant predictors for the survival of the patients who received with S‐1 as adjuvant chemotherapy agent (Table 3).

**TABLE 3:** Prognostic factors for the overall survival in the 95 patients classified by the kind of adjuvant agents (GEM or S‐1)

Variables	Univariate		Multivariate	
	Hazard ratio (95% confidence interval)	p	Hazard ratio (95% confidence interval)	p
GEM (*n* = 48)				
Sex (Male/Female)	1.90 (1.03–3.50)	0.041	1.90 (1.03–3.51)	0.040
Age (≥65 years/<65 years)	1.13 (0.61–2.10)	0.698		
ECOG performance status (1/0)	1.19 (0.64–2.23)	0.585		
Residual tumor status (R1/R0)	1.62 (0.79–3.32)	0.190		
Regional lymph node status (N1/N0)	1.21 (0.63–2.33)	0.570		
CA19‐9 (>37 U/ml/≤37 U/ml)	1.00 (0.51–1.97)	0.993		
hENT1 expressions				
TMA with 10D7G2 (High/Low)	0.26 (0.08–0.84)	0.025	0.24 (0.08–0.84)	0.024
S‐1 (*n* = 47)				
Sex (Male/Female)	0.75 (0.36–1.58)	0.449		
Age (≥65 years/<65 years)	1.32 (0.63–2.74)	0.464		
ECOG performance status (1/0)	1.12 (0.52–2.42)	0.767		
Residual tumor status (R1/R0)	1.74 (0.60–5.00)	0.307		
Regional lymph node status (N1/N0)	1.32 (0.54–3.24)	0.546		
CA19‐9 (>37 U/ml/≤37 U/ml)	2.15 (0.99–4.67)	0.053		
hENT1 expressions				
TMA with 10D7G2 (High/Low)	0.71 (0.27–1.86)	0.483		

Abbreviations: ECOG, Eastern Cooperative Oncology Group; GEM, gemcitabine; TMA, tissue microarray.

### 3.5 Overall survival by SP120 hENT1 expression

Among the 326 patients whose IHC with SP120 were evaluable (Figure 1(A),(C)–(E)), the median survival times of patients treated with GEM who showed absent, weak, moderate and strong IHC with SP120 were 2.09, 2.19, 2.08 and 2.13 years, respectively (Figure S4(A)). There were no significant differences among the four groups according to IHC with SP120. Among patients treated with GEM, those with SP120^High^ showed an equivalent survival compared with those with SP120^Low^ (median 2.13 vs. 2.16 years, HR [SP120^High^] 0.78, 95% CI 0.42–1.45, p = .429, Figure 2(C)).

The median survival times of patients treated with S‐1 who showed absent, weak, moderate and strong IHC with SP120 were: “not reached” due to 5‐year overall survival rate >50%, 4.49, 2.81 and 2.56 years, respectively (Figure S4(B)). As we previously reported,^20^ there was a significant difference in overall survival between patients with SP120^Low^ and SP120^High^ when the cut‐off was set between absent/weak and moderate/strong, and multivariate analysis showed that SP120^High^ (HR 1.61, 95% CI 1.06–2.45, p = .027) was one of significant predictors for the survival of the patients who received S‐1 as adjuvant chemotherapy. However, when the cut‐off was set between absent/weak/moderate and strong, which showed the highest concordance with TMA with 10D7G2 Ab (Table 2), those with SP120^Low^ showed substantially longer survival compared with those with SP120^High^ (median 3.86 versus 2.56 years, HR [SP120^Low^] 0.66, 95% CI 0.35–1.24, p = .196, Figure 2(D)). However, multivariate analysis showed that SP120^High^ whose cut‐off set between moderate and strong was marginally not a significant prognostic factor of patients treated with S‐1 (Table 4).

**TABLE 4:** Prognostic factors for the overall survival in the 326 patients classified by the kind of adjuvant agents (GEM or S‐1)

Variables	Univariate		Multivariate	
	Hazard ratio (95% confidence interval)	p	Hazard ratio (95% confidence interval)	p
GEM (*n* = 166)				
Sex (Male/Female)	1.24 (0.88–1.75)	0.219		
Age (≥65 years/<65 years)	1.12 (0.78–1.60)	0.540		
ECOG performance status (1/0)	1.46 (1.02–2.09)	0.037	1.29 (0.89–1.87)	0.177
Residual tumor status (R1/R0)	1.99 (1.24–3.20)	0.004	1.48 (0.91–2.41)	0.117
Primary tumor status (T3‐T4/T1‐2)	4.22 (1.97–9.07)	<0.001	3.40 (1.56–7.42)	0.002
Regional lymph node status (N1/N0)	1.89 (1.30–2.75)	0.001	1.35 (0.90–2.01)	0.143
CA19‐9 (>37 U/ml/≤37 U/ml)	1.84 (1.24–2.73)	0.003	1.54 (1.02–2.32)	0.040
hENT1 expressions				
IHC with SP120 (Strong/Moderate+Weak+Absent)	0.78 (0.42–1.45)	0.430		
IHC with SP120 (Strong+Moderate/Weak+Absent)	0.95 (0.65–1.38)	0.786		
IHC with SP120 (Strong+Moderate+Weak/Absent)	1.01 (0.68–1.50)	0.952		
S‐1 (*n* = 160)				
Sex (Male/Female)	1.07 (0.72–1.61)	0.728		
Age (≥65 years/<65 years)	1.19 (0.79–1.79)	0.404		
ECOG performance status (1/0)	1.08 (0.70–1.67)	0.716		
Residual tumor status (R1/R0)	1.89 (1.10–3.23)	0.020	1.90 (1.11–3.27)	0.020
Primary tumor status (T3‐T4/T1‐2)	1.50 (0.73–3.10)	0.269		
Regional lymph node status (N1/N0)	2.02 (1.26–3.25)	0.004	1.83 (1.13–2.97)	0.014
CA19‐9 (>37 U/ml/≤37 U/ml)	2.11 (1.35–3.29)	0.001	2.02 (1.29–3.17)	0.002
hENT1 expressions				
IHC with SP120 (Strong/Moderate+Weak+Absent)	1.51 (0.81–2.83)	0.200		
IHC with SP120 (Strong+Moderate/Weak+Absent)	1.75 (1.16–2.64)	0.008	1.61 (1.06–2.45)	0.027
IHC with SP120 (Strong+Moderate+Weak/Absent)	1.65 (0.98–2.78)	0.061		

Abbreviations: ECOG, Eastern Cooperative Oncology Group; GEM, gemcitabine; hENT1, human equilibrative nucleoside transporter‐1; IHC, immunohistochemistry.

### 3.6 *hENT1* mRNA expression and overall survival

Among 310 patients whose *hENT1* mRNA levels were available (Figure 1(C),(D)), the area under the curve (AUCs) values of the *hENT1* mRNA level was 0.524 for predicting 2‐year overall survival (Figure S5(A)) and 0.531 for predicting 3‐year overall survival (Figure S5(B)). Because these AUCs were lower than 0.7, it was difficult to determine the optimal cut‐off values of the *hENT1* mRNA levels for predicting survival.

### 3.7 Treatment selection according to hENT1 expression

Significance of hENT1 expression with 10D7G2 for selecting adjuvant chemotherapy with GEM or S‐1 was investigated in the 95 patients (Figure 1(A)–(C)), and that with SP120, with the cut‐off set between moderate and strong (the highest concordance rate between 10D7G2 Ab and SP120 Ab), was done in 326 patients (Figure 1(A)–(E)).

Similarly to the overall population in this study (Figure S2(A)), the overall survival of the S‐1‐treated groups was significantly better than that of the GEM‐treated group both in 10D7G2^Low^ (*n* = 79, Figure S6(A)) and SP120^Low^ (*n* = 295, Figure S6(C)) subgroups, and multivariate analyses showed that adjuvant chemotherapy with GEM was a significant predictor for poor survival of the patients in both subgroups (Table 5).

**TABLE 5:** Prognostic factors for the prognosis in the patients classified by the hENT1 expression by each antibody

Variables	Univariate		Multivariate	
	Hazard ratio (95% Confidence interval)	p	Hazard ratio (95% Confidence interval)	p
The patients with 10D7G2^Low^ (*n* = 79)				
Residual tumor status (R1/R0)	1.81 (0.96–3.41)	0.067	1.67 (0.88–3.18)	0.116
Adjuvant agent (GEM/S‐1)	2.44 (1.45–4.12)	0.001	2.39 (1.41–4.06)	0.001
The patients with 10D7G2^High^ (*n* = 16)				
CA19‐9 (>37 U/ml/≤37 U/ml)	4.77 (0.79–28.9)	0.089	4.86 (0.79–29.8)	0.088
Adjuvant agent (GEM/S‐1)	0.94 (0.22–3.94)	0.931	1.17 (0.26–5.37)	0.931
The patients with SP120^Low^ (*n* = 295)				
Residual tumor status (R1/R0)	2.35 (1.63–3.38)	<0.001	1.88 (1.30–2.74)	0.001
Primary tumor status (T3‐T4/T1‐2)	2.64 (1.50–4.63)	0.001	2.14 (1.19–3.85)	0.011
Regional lymph node status (N1/N0)	1.83 (1.35–2.48)	<0.001	1.50 (1.08–2.07)	0.015
CA19‐9 (>37 U/ml/≤37 U/ml)	1.87 (1.37–2.57)	<0.001	1.59 (1.15–2.20)	0.005
Adjuvant agent (GEM/S‐1)	1.73 (1.31–2.28)	<0.001	1.84 (1.39–2.44)	<0.001
The patients with SP120^High^ (*n* = 31)				
ECOG performance status (1/0)	2.60 (1.08–6.27)	0.033	2.75 (1.03–7.33)	0.044
CA19‐9 (>37 U/ml/≤37 U/ml)	2.81 (1.19–6.64)	0.018	2.47 (0.50–3.35)	0.047
Adjuvant agent (GEM/S‐1)	0.93 (0.40–2.16)	0.872	0.77 (0.30–2.00)	0.592

Abbreviations: GEM, gemcitabine; hENT1, human equilibrative nucleoside transporter‐1.

In contrast, the overall survival between the GEM‐ and S‐1‐treated groups was not significantly different either in 10D7G2^High^ (*n* = 16, Figure S6(B)) and SP120^High^ (*n* = 31, Figure S6(D)) subgroups. Moreover, multivariate analysis showed that agents for adjuvant chemotherapy, GEM or S‐1, was not a significant predictor for the prognosis of the patients with high hENT1 expression regardless of the used Ab, either 10D7G2 (*n* = 16) or SP120 (*n* = 31) (Table 5).

## 4 DISCUSSION

The patients' clinical and pathological factors in the present study was not much different from those in the JASPAC 01 study,^20^ and the all subjects of this study recapitulated the clinical outcomes in JASPAC 01 study. Moreover, the freshness of the specimens in this study is much better than those of the earlier three studies,^14^, ^18^, ^19^ as the present study used specimens from a randomized controlled trial performed during short period (3 years). From these facts, it is considered that the present study have a certain degree of quality.

The present study showed no correlations between the *hENT1* mRNA level and hENT1 expressions using either 10D7G2 or SP120 Ab and suggests that the concordance rate between the two Abs for evaluating hENT1 expression depends on the cut‐off point set among four groups according to IHC staining with SP120 Ab. However, there were several problems in the studies focusing on this issue. First, the platform between the two Abs, IHC and TMA, was different. Second, even among studies that used 10D7G2 Ab, some studies evaluated hENT1 expression with H‐scores,^11^, ^18^, ^23^ and other studies categorized samples into three or four groups according to tumor staining intensity.^8^, ^10^, ^13^, ^14^, ^19^, ^23^ The two Abs should be compared based on the same assessment methods.

H‐scores are obtained as continuous values, and cut‐off values for high and low expression, to predict sensitivity to GEM, were different among studies. In earlier studies which used the median H‐score as their cut‐off value, median H‐scores (48^11^ and 90^18^) varied even using same Ab (10D7G2). The median H‐score of the present study was 100, which was higher than that of the earlier studies,^11^, ^18^ and the cut‐off value of H‐scores (135) was determined based on the minimum p value approach to predict survival. If the cut‐off value in the present study had been set at the median H‐score, it would not show the patients with 10D7G2^High^ to derive any survival benefit from adjuvant chemotherapy with GEM. In the systemic review about the utility of hENT1 expression to predict sensitivity to GEM, the positive rate of hENT1 expression varied from 39% to 80% (10D7G2).^23^

On the other hands, almost all studies using SP120 Ab^14^, ^15^, ^16^, ^17^, ^19^, ^23^ evaluated hENT1 expression by classifying into 2–4 groups according to tumor staining intensity except one study.^18^ The positive rates of hENT1 expression by SP120 Ab varied from 21% to 72%, similarly to those by 10D7G2 Ab.^14^, ^15^, ^16^, ^17^, ^18^, ^19^, ^23^ Compared to earlier studies, the positive rate of hENT1 expression with SP120 Ab in the present study was extremely low (11%) when the cut‐off between SP120^Low^ and SP120^High^ was set between moderate and strong, which showed the highest concordance with TMA with 10D7G2 Ab. However, with cut‐off set between weak and moderate, the positive rate will be compatible to those of previous studies. Therefore, the utility of hENT1 expression in predicting survival will differ depending on the cut‐off value regardless the platform and Abs for its evaluation.

We used the different measurement platforms for hENT1 expression with two Abs (TMA for 10D7G2 and IHC for SP120) in the present study. Expression of hENT1 as assessed by both 10D7G2 and SP120 Abs was matched in 79.8% when the cut‐off was set between moderate and strong, leading to a low positive rate. The concordance rate was higher than those of earlier studies (59.7%, 69.1% and 50.7%).^14^, ^18^, ^19^ However, the concordance rate in this study decreased when the cut‐off was set between weak and moderate, leading to a higher positive rate. Many of studies that examined specimens from large‐ scale clinical trials used TMAs with small cores from the FFPE specimens.^8^, ^9^, ^10^, ^11^, ^14^, ^15^, ^16^, ^17^, ^18^, ^19^ However, as PC tumors are very heterogeneous, the results obtained from TMA might have been affected by the collected parts of the tumors. It is speculated that the hENT1 expression may be underestimated using TMA than IHC in which the part with the strongest staining would draw attention.

The inconsistency between immunohistochemical expression of hENT1 (for both 10D7G2 and SP120) and *hENT1* mRNA level was another important issue. In the present study, we analyzed expression levels of both hENT1 using two kind of Ab and *hENT1* mRNA level. To our knowledge, no other studies had compared immunohistochemical expression of hENT1 with *hENT1* mRNA levels in PC tissues, but Raffenne J et al. showed the correlation between the hENT1 expression evaluated by 10D7G2 and *hENT1* mRNA levels during preparing this manuscript.^14^ Unlike the results of Raffenne J et al.,^14^ as one reason why these two expression levels were inconsistent in the current study, macro‐dissection to extract the samples for evaluating *hENT1* mRNA level might include components other than cancer cells because PC with a strongly tendency to have stromal tissues. Considering that there were low association between mRNA level and survival in spite of some relation with IHC and TMA, there might be unknown process from the mRNA level to the protein level^24^ especially in PC.

Considering the actual clinical practice for selecting the adjuvant chemotherapy agent, the present study suggests 10D7G2 Ab is more useful to predict the overall survival than SP120 limited in the GEM‐treated patients. In the subgroup analyses according to hENT1 expression by each 10D7G2 Ab and SP120, introducing GEM as adjuvant agent was significant unfavorable predictor for survival both in 10D7G2^Low^ and SP120^Low^ groups, which suggests that S‐1 not GEM should be introduced for the patients with low hENT1 expression no matter which Ab is used. In contrast, the kind of adjuvant agent was not significant predictor for survival in both 10D7G2^High^ and SP120^High^ groups, which suggests that either S‐1 or GEM can be introduced for the patients with high hENT1 expression no matter which Ab is used.

From another view by the kind of adjuvant agent, 10D7G2^High^ was a significant favorable predictor for the survival in the GEM‐treated patients, and the overall survival rate of the 10D7G2^High^ group was significantly better than that of the 10D7G2^Low^ group; 5‐year overall survival for the GEM‐treated 10D7G2^High^ group was 41.7%, which was similar to the survival rate for patients treated with adjuvant S‐1 in JASPAC 01 study,^20^ even though the rate of N1 regional lymph nodes was marginally higher in the 10D7G2^High^ group. This result may suggest that GEM, rather than S‐1, is the better choice for adjuvant chemotherapy among patients with 10D7G2^High^.

Now, there are several options for adjuvant chemotherapy (FOLFIRINOX,^25^ capecitabine,^4^ S‐1^20^ or GEM^26^) and introducing either regimen has higher priority than GEM as the adjuvant agent for PC. Giving the current treatment strategy for PC, the opportunity to use GEM as the adjuvant agent for the first choice is quite low and the benefit of 10D7G2 use may be also low at the same time.

The present study has several limitations. This biomarker study was designed retrospectively, after completing the final analysis of the JASPAC 01 study. Especially, collecting the FFPE specimens from participating institutions was difficult. As a result, the concordance of hENT1 expression between the two Abs was investigated in only 89 patients. Although TMAs cannot be constructed without FFPE specimens, we could collect the unstained sections.^20^, ^21^ If we could obtain 10D7G2 Ab for IHC, we could have validated its worth in larger cohort. Moreover, all patients who enrolled in the JASPAC 01 study were East Asian. The pharmacokinetics and pharmacodynamics of anti‐cancer drugs in European and North American patients and patients from East Asia might differ due to genetic differences.

In conclusion, the present study suggests that the two Abs for evaluating hENT1 expression are equivalent depending on the cut‐off point. No matter which antibody is used, S‐1 is the first choice of adjuvant chemotherapy for the PC patients with low hENT1 expression, whereas either S‐1 or GEM can be introduced for the PC patients with high hENT1 expression.

## CONFLICT OF INTEREST

Katsuhiko Uesaka received honoraria from Taiho. Narikazu Boku received honoraria from Taiho and Eli Lilly. Akira Fukutomi received honoraria from Taiho, Eli Lilly Japan, Yakult Honsha, and Daiichi Sankyo; and received rewards for an advisory role from Yakult Honsha.

## AUTHORS' CONTRIBUTIONS

All authors had full access to the data in the study and take responsibility for the integrity of the data and the accuracy of the data analysis. *Conceptualization*, Y.O., B.N., G.P., G.W., Y.S., N.H., F.A., K.M., M.S., T.H., M.A., S.Y., N.S., S.N., Y.K., T.A., T.W., Y.R., T.M., Y.A., N.P.J. and U.K.; *Methodology*, Y.O., B.N., G.P., G.W., Y.S., N.H., F.A., K.M., M.S., T.H., M.A., S.Y., N.S., S.N., Y.K., T.A., T.W., Y.R., T.M., Y.A., N.P.J. and U.K.; *Investigation*, Y.O., B.N., G.P., G.W., Y.S., N.H., F.A., K.M., M.S., T.H., M.A., S.Y., N.S., S.N., Y.K., T.A., T.W., Y.R., T.M., Y.A., N.P.J. and U.K.; *Formal Analysis*, Y.O., B.N., G.P., G.W., Y.S., N.H., F.A., K.M., M.S., T.H., M.A., S.Y., N.S., S.N., Y.K., T.A., T.W., Y.R., T.M., Y.A., N.P.J. and U.K.; *Resources*, Y.O., B.N., G.P., G.W., Y.S.,N.H., F.A., K.M., M.S., M.A., Y.R., Y.A., N.P.J. and U.K.; *Writing‐Original Draft*, Y.O., B.N., G.P., G.W., Y.S., N.H., F.A., K.M., M.S., T.H., M.A., S.Y., N.S., S.N., Y.K., T.A., T.W., Y.R., T.M., Y.A., N.P.J. and U.K.; *Writing‐Review Editing*, Y.O., B.N., G.P., G.W., Y.S., N.H., F.A., K.M., M.S., T.H., M.A., S.Y., N.S., S.N., Y.K., T.A., T.W., Y.R., T.M., Y.A., N.P.J. and U.K.; *Visualization*, Y.O., B.N., G.P., G.W., Y.S., N.H., F.A., K.M., M.S., T.H., M.A., S.Y., N.S., S.N., Y.K., T.A., T.W., Y.R., T.M., Y.A., N.P.J. and U.K.; *Supervision*, Y.O., B.N., G.P., G.W., Y.S., N.H., F.A., K.M., M.S., T.H., M.A., S.Y., N.S., S.N., Y.K., T.A., T.W., Y.R., T.M., Y.A., N.P.J. and U.K.; *Funding Acquisition*, Y.O., B.N., G.P., G.W., Y.S., N.H., F.A., K.M., M.S., T.H., M.A., S.Y., N.S., S.N., Y.K., T.A., Y.R., T.M., Y.A., N.P.J. and U.K.; *Data Curation*, Y.O., B.N.,G.P., G.W., Y.S., N.H., F.A., K.M., M.S., T.H., M.A., S.Y., N.S., S.N., Y.K., T.A., T.W., Y.R., T.M., Y.A., N.P.J. and U.K.; *Project Administration*, Y.O., B.N., G.P., G.W., Y.S., N.H., F.A., K.M., M.S., T.H., M.A., S.Y., N.S., S.N., Y.K., T.A., T.W., Y.R., T.M., Y.A., N.P.J. and U.K.; *Software*, Y.O., B.N., G.P., G.W., Y.S., N.H., F.A., K.M., M.S., T.H., M.A., S.N., Y.A., N.P.J. and U.K.; *Validation*, Y.O., B.N., G.P., G.W., Y.S., N.H., F.A., K.M., M.S., T.H., M.A., S.Y., N.S., S.N., Y.K., T.A., T.W., Y.R., T.M., Y.A., N.P.J. and U.K.

## ETHICAL STATEMENTS

The protocol of the present study was approved by the ethics committee of the Shizuoka Cancer Center (No. 27‐22‐27‐1‐5) and the institutional review board of each participating institution. Written informed consent for this study was obtained as required by the Institutional Review Board.

## Supporting information


**Figure S1.** Examples of cases with the intensity of immunohistochemistry staining of cytoplasmic human equilibrative nucleoside transporter‐1 (hENT1) expression using the SP 120 antibody. In the scale bar, 100 showed 100 μm.
A: no, B: weak, C: moderate, D: strong.

**Figure S2.** A: Kaplan–Meier curves for overall survival of the patients in the present study.
B: Kaplan–Meier curves for relapse‐free survival of the patients in the present study.

**Figure S3.** Correlation between human equilibrative nucleoside transporter‐1 (hENT1) expression levels (H‐scores by 10D7G2 antibody [A] and intensity of immunostaining by SP120 antibody [B]) and mRNA expression levels.

**Figure S4.** A: Kaplan–Meier curves for overall survival in the gemcitabine arm, stratified by human equilibrative nucleoside transporter‐1 (hENT1) expression by SP120.
B: Kaplan–Meier curves for overall survival in the S‐1 arm, stratified by hENT1 expression by SP120.

**Figure S5.** Receiver operating characteristic curves of *hENT1* mRNA levels for predicting 2‐year overall survival (A) and for predicting 3‐year overall survival (B).

**Figure S6.** A: Kaplan–Meier curves for overall survival in the 10D7G2^Low^ group, stratified by adjuvant chemotherapy agent.
B: Kaplan–Meier curves for overall survival in the 10D7G2^High^ group, stratified by adjuvant chemotherapy agent.
C: Kaplan–Meier curves for overall survival in the SP120^Low^ group, stratified by adjuvant chemotherapy agent.
D: Kaplan–Meier curves for overall survival in the SP120^High^ group, stratified by adjuvant chemotherapy agent.

**Table S1.** The clinical and pathological characteristics of the patients whose H‐score by TMA with 10D7G2 Ab were evaluable.

## Data Availability

Author elects to not share data.
