# Supplementary figures and images for: Concordance of human equilibrative nucleoside transporter‐1 expressions between murine (10D7G2) and rabbit (SP120) antibodies and association with clinical outcomes of adjuvant chemotherapy for pancreatic cancer: A collaborative study from the JASPAC 01 trial
Source: Cancer Rep (Hoboken). 2021 Jul 29;5(5):e1507. doi: 10.1002/cnr2.1507 (PMC9124504; doi:10.1002/cnr2.1507)

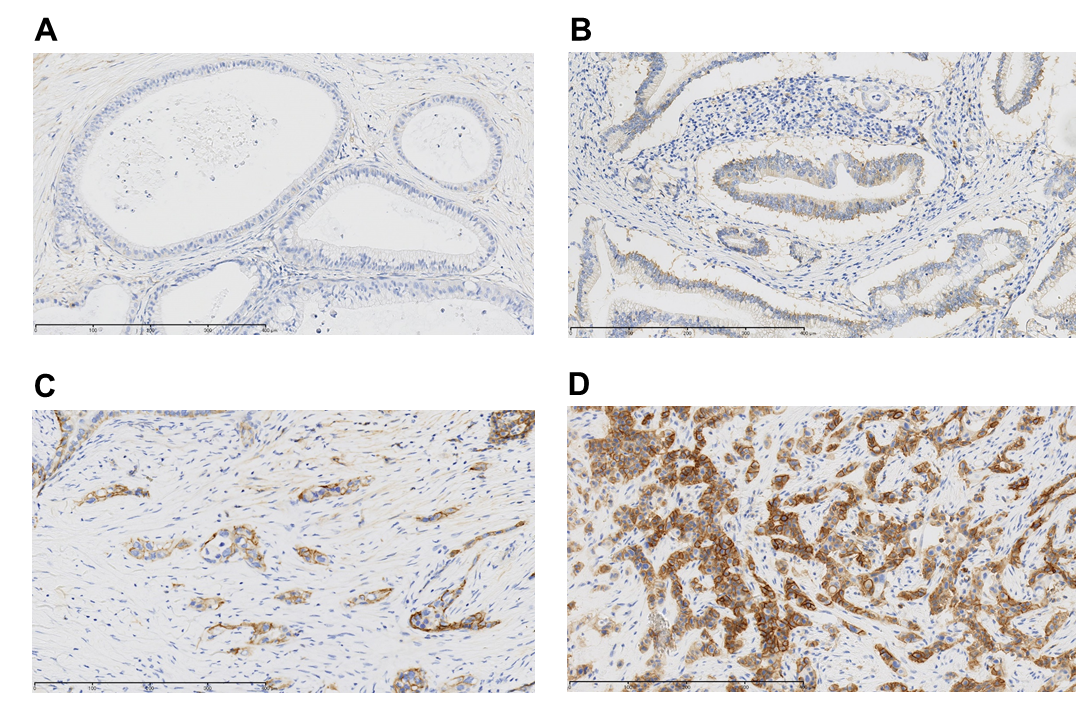

Supplement: Supplementary file 1 — Figure S1. Examples of cases with the intensity of immunohistochemistry staining of cytoplasmic human equilibrative nucleoside transporter‐1 (hENT1) expression using the SP 120 antibody. In the scale bar, 100 showed 100 μm. A: no, B: weak, C: moderate, D: strong. [file CNR2-5-e1507-s001.tif]

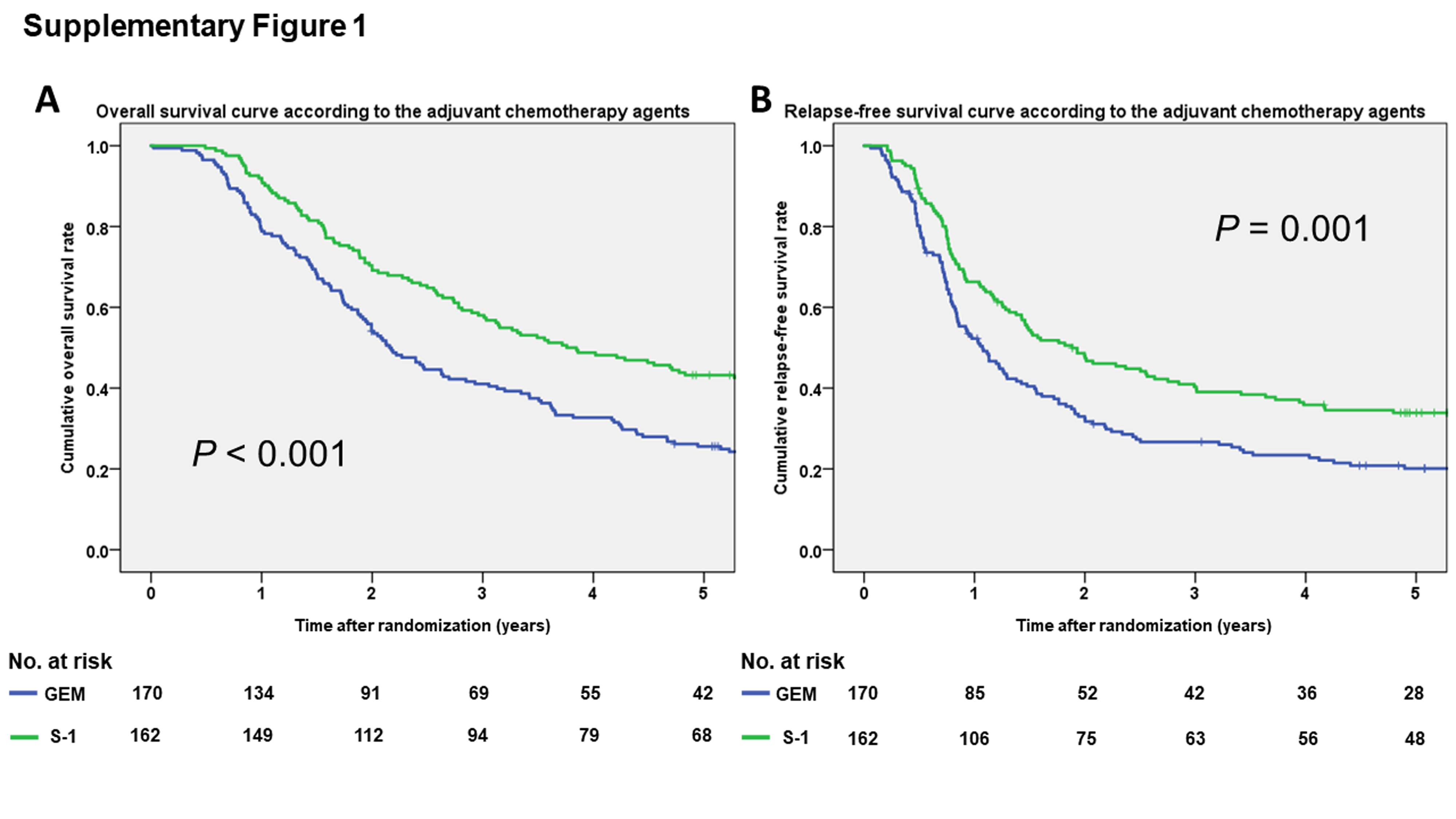

Supplement: Supplementary file 2 — Figure S2. A: Kaplan–Meier curves for overall survival of the patients in the present study. B: Kaplan–Meier curves for relapse‐free survival of the patients in the present study. [file CNR2-5-e1507-s002.tif]

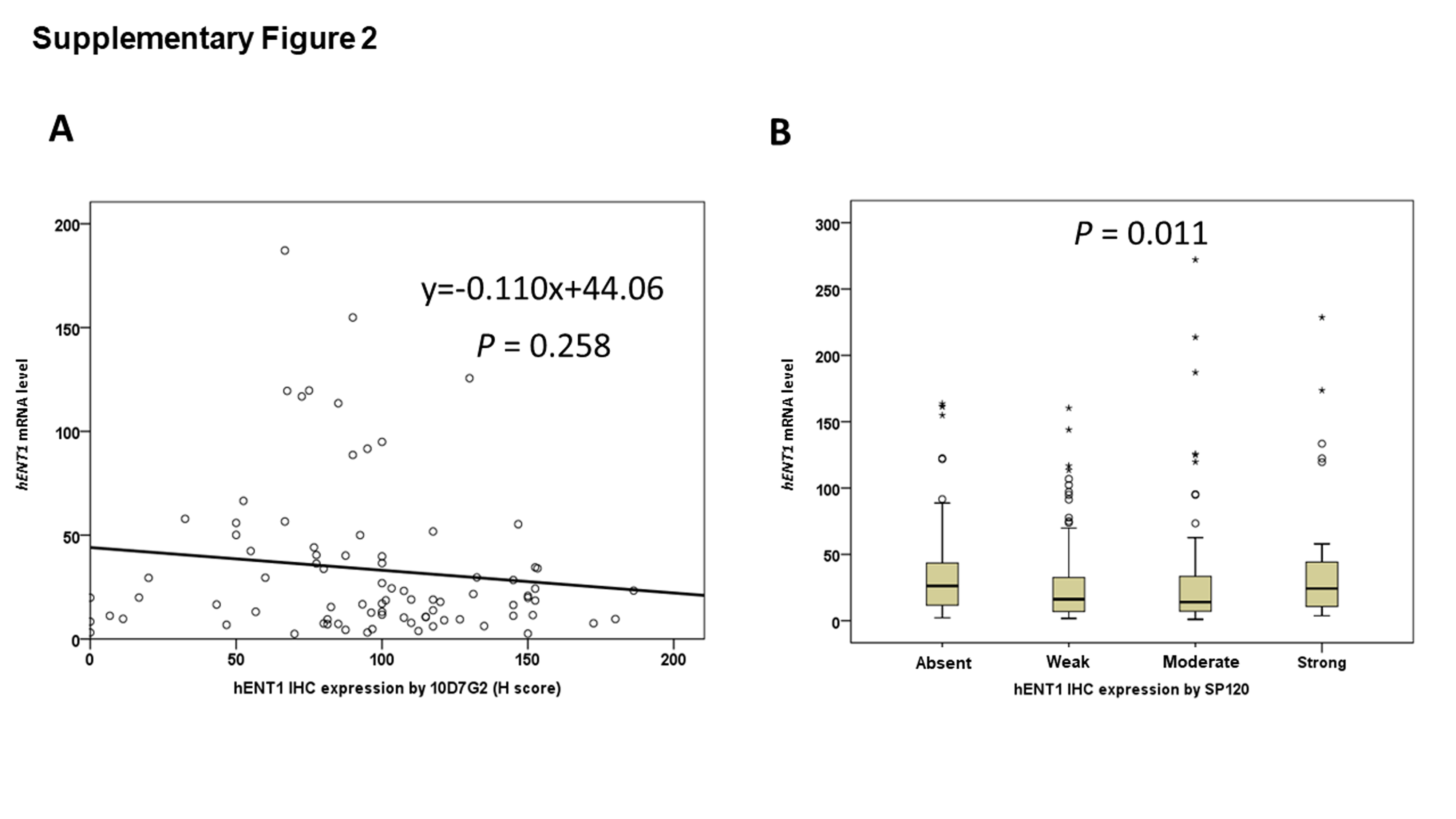

Supplement: Supplementary file 3 — Figure S3. Correlation between human equilibrative nucleoside transporter‐1 (hENT1) expression levels (H‐scores by 10D7G2 antibody [A] and intensity of immunostaining by SP120 antibody [B]) and mRNA expression levels. [file CNR2-5-e1507-s004.tif]

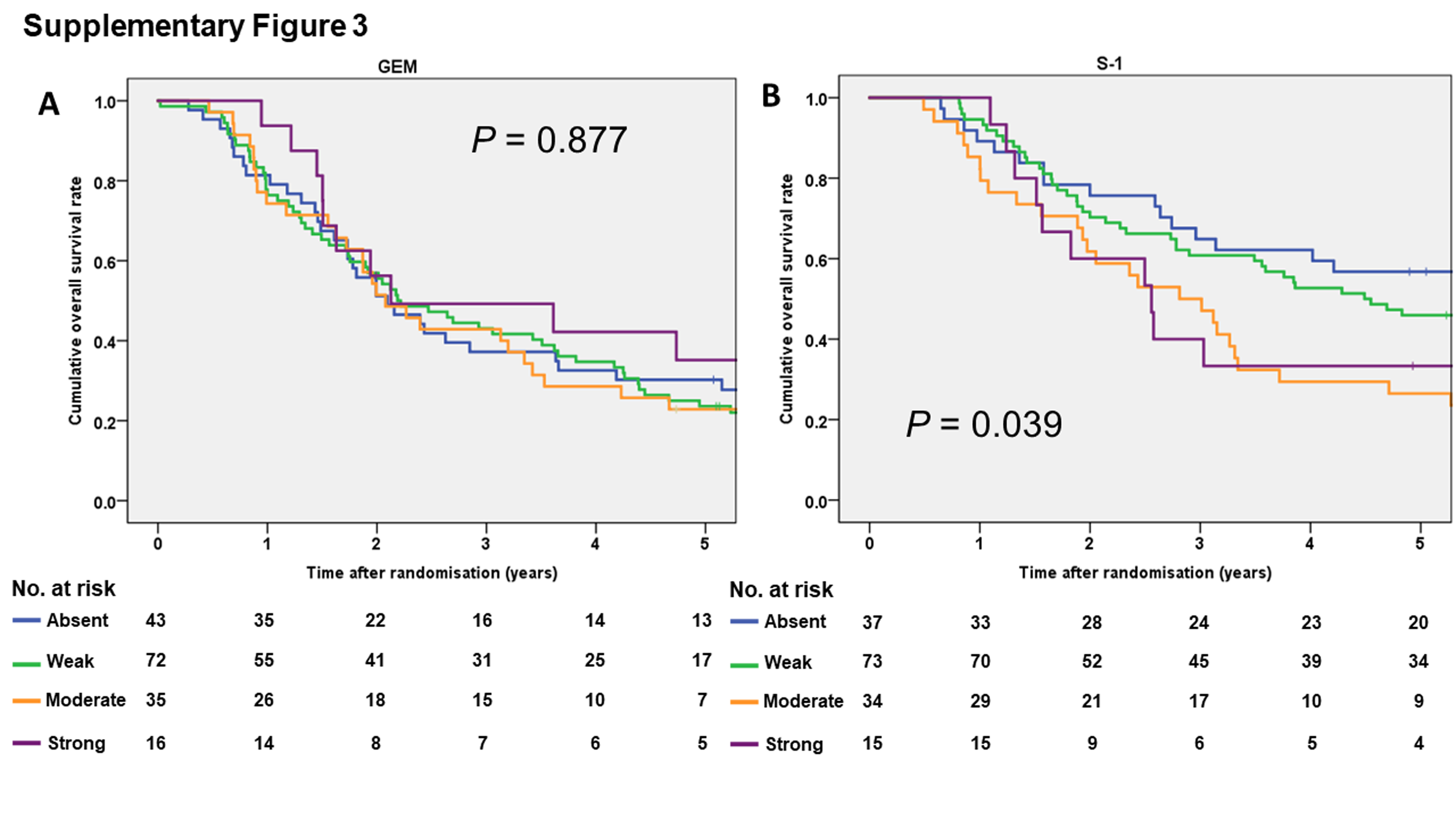

Supplement: Supplementary file 4 — Figure S4. A: Kaplan–Meier curves for overall survival in the gemcitabine arm, stratified by human equilibrative nucleoside transporter‐1 (hENT1) expression by SP120. B: Kaplan–Meier curves for overall survival in the S‐1 arm, stratified by hENT1 expression by SP120. [file CNR2-5-e1507-s006.tif]

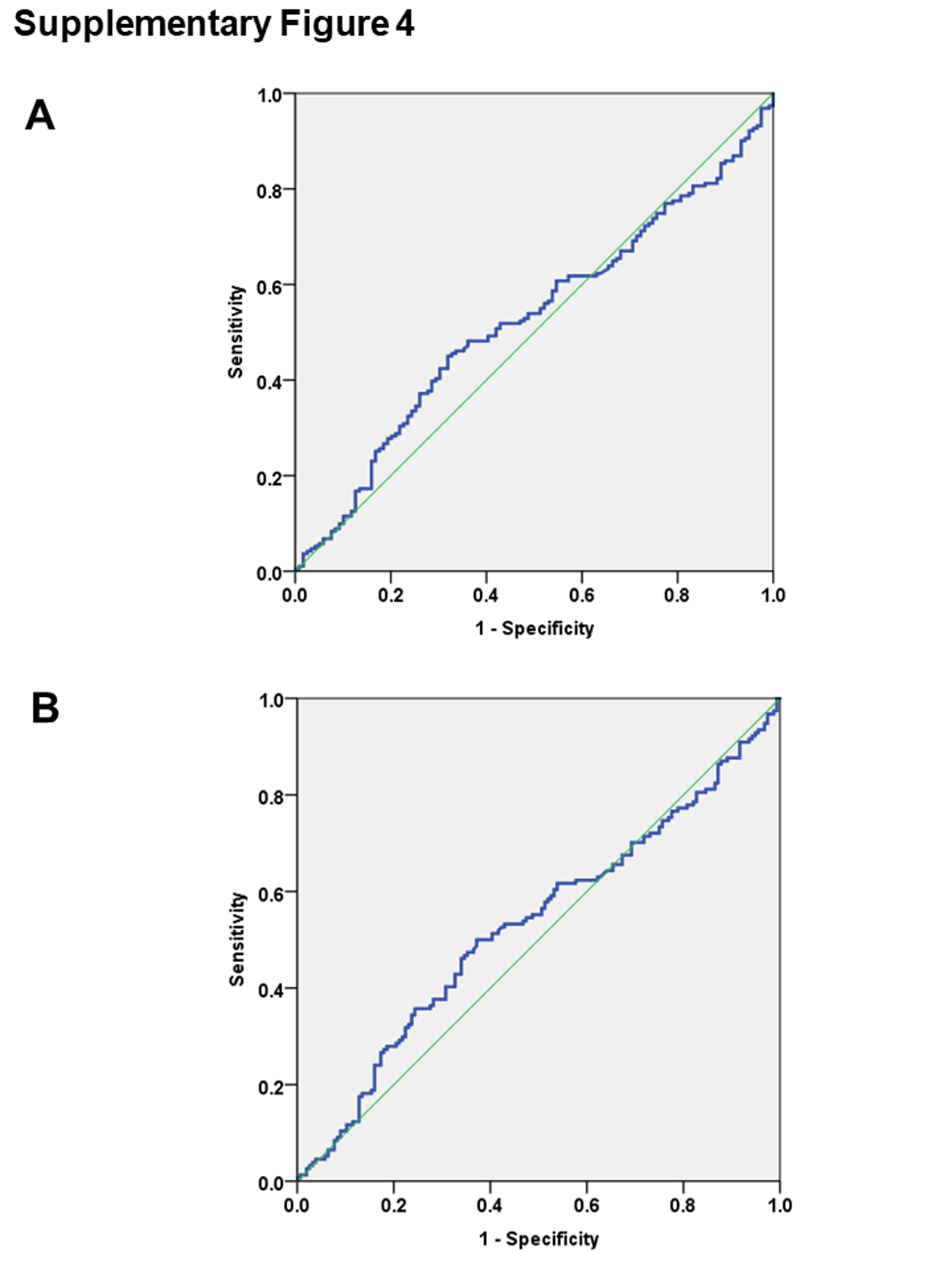

Supplement: Supplementary file 5 — Figure S5. Receiver operating characteristic curves of hENT1 mRNA levels for predicting 2‐year overall survival (A) and for predicting 3‐year overall survival (B). [file CNR2-5-e1507-s003.tif]

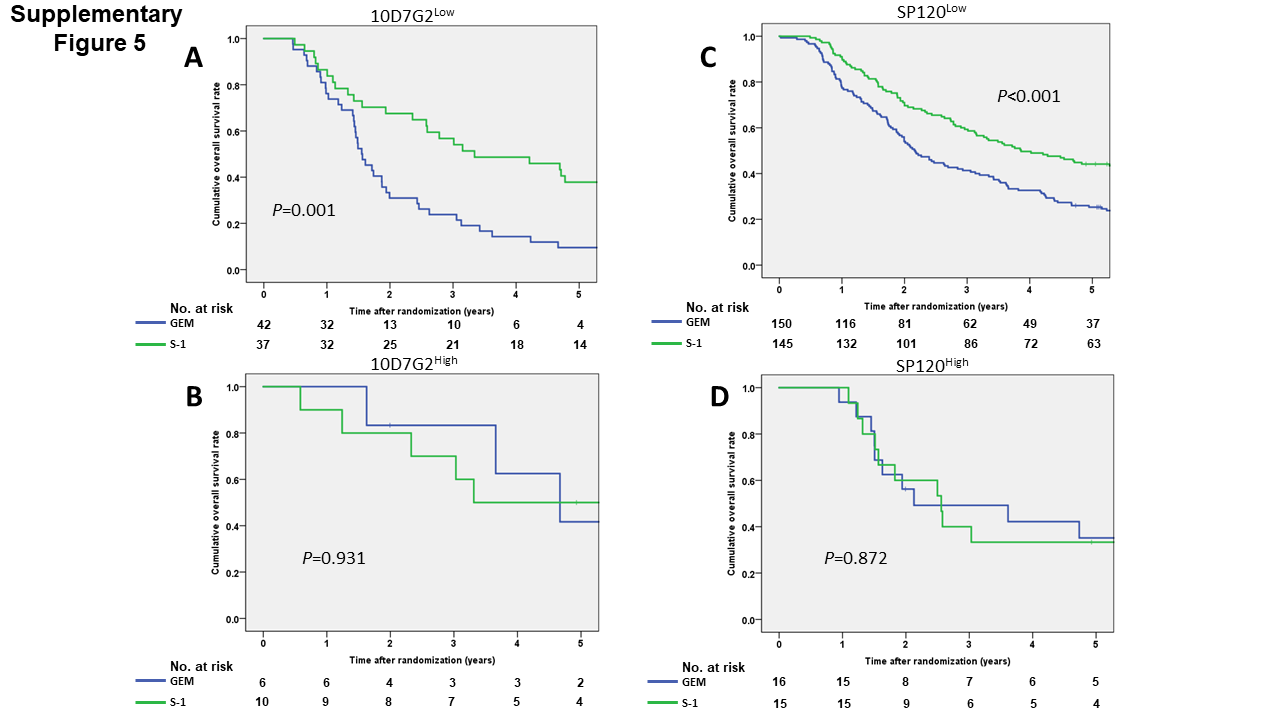

Supplement: Supplementary file 6 — Figure S6. A: Kaplan–Meier curves for overall survival in the 10D7G2Low group, stratified by adjuvant chemotherapy agent. B: Kaplan–Meier curves for overall survival in the 10D7G2High group, stratified by adjuvant chemotherapy agent. C: Kaplan–Meier curves for overall survival in the SP120Low group, stratified by adjuvant chemotherapy agent. D: Kaplan–Meier curves for overall survival in the SP120High group, stratified by adjuvant chemotherapy agent. [file CNR2-5-e1507-s007.tif]
