# Supplementary material for: Concordance of human equilibrative nucleoside transporter‐1 expressions between murine (10D7G2) and rabbit (SP120) antibodies and association with clinical outcomes of adjuvant chemotherapy for pancreatic cancer: A collaborative study from the JASPAC 01 trial
Source: Cancer Rep (Hoboken). 2021 Jul 29;5(5):e1507. doi: 10.1002/cnr2.1507 (PMC9124504; doi:10.1002/cnr2.1507)
Supplement: Supplementary file 7 — Table S1. The clinical and pathological characteristics of the patients whose H‐score by TMA with 10D7G2 Ab were evaluable. [file CNR2-5-e1507-s005.docx]

| **Supplementary Table 1** The clinical and pathological characteristics of the patients whose H-score by TMA with 10D7G2 Ab were evaluable | | | | | | |
| --- | --- | --- | --- | --- | --- | --- |
|  | Gemcitabine (n=48) | |  | S-1 (n=47) | |  |
|  | 10D7G2^high^ (n=6) | 10D7G2^Low^  (n=42) | *P* | 10D7G2^high^  (n=10) | 10D7G2^Low^  (n=37) | *P* |
| Sex |  |  | 0.667 |  |  | 1.000 |
| Male | 2 (33) | 22 (52) |  | 6 (60) | 23 (62) |  |
| Female | 4 (67) | 20 (48) |  | 4 (40) | 14 (38) |  |
| Age* | 60 (57–68) | 68 (44–78) | 0.276 | 63 (50–78) | 65 (36–86) | 1.000 |
| ECOG performance status |  |  | 1.000 |  |  | 0.716 |
| 0 | 4 (67) | 26 (62) |  | 6 (60) | 25 (68) |  |
| 1 | 2 (33) | 16 (38) |  | 4 (40) | 12 (32) |  |
| Residual tumor status |  |  | 1.000 |  |  | 0.594 |
| R0 | 5 (83) | 32 (76) |  | 8 (80) | 33 (89) |  |
| R1 | 1 (17) | 10 (24) |  | 2 (20) | 4 (11) |  |
| Primary tumor status^**^ |  |  | 0.336 |  |  | 0.384 |
| T1–T2 | 1 (17) | 2 (5) |  | 1 (10) | 1 (3) |  |
| T3–T4 | 5 (83) | 40 (95) |  | 9 (90) | 36 (97) |  |
| Regional lymph node status^**^ |  |  | 0.402 |  |  | 0.088 |
| N0 | 1 (17) | 16 (38) |  | 0 (0) | 11 (30) |  |
| N1 | 5 (83) | 26 (62) |  | 10 (100) | 26 (70) |  |
| CA19-9 |  |  | 1.000 |  |  | 0.409 |
| <37 U/mL | 5 (83) | 30 (71) |  | 9 (90) | 26 (70) |  |
| >37 U/mL | 1 (17) | 12 (29) |  | 1 (10) | 11 (30) |  |
| Pathological stage^**^ |  |  | 0.639 |  |  | 0.088 |
| IA | 0 (0) | 0 (0) |  | 0 (0) | 0 (0) |  |
| IB | 0 (0) | 1 (2) |  | 0 (0) | 0 (0) |  |
| IIA | 1 (17) | 14 (33) |  | 0 (0) | 11 (30) |  |
| IIB | 5 (83) | 27 (65) |  | 10 (100) | 26 (70) |  |
| III | 0 (0) | 0 (0) |  | 0 (0) | 0 (0) |  |
| TMA: Tissue microarray; Ab: antibody; ECOG: Eastern Cooperative Oncology Group.  Values in parentheses are percentages unless indicated otherwise; *value is expressed as the median (range).  **Primary tumor status, regional lymph node status, and pathological stage according to the TNM Classification of Malignant Tumours, 6th edition. | | | | | | |
